# Field testing a novel high residence positioning system for monitoring the fine‐scale movements of aquatic organisms

**DOI:** 10.1111/2041-210X.12993

**Published:** 2018-03-24

**Authors:** Matthew M. Guzzo, Travis E. Van Leeuwen, Jack Hollins, Barbara Koeck, Matthew Newton, Dale M. Webber, Frank I. Smith, David M. Bailey, Shaun S. Killen

**Affiliations:** ^1^ Department of Biological Sciences University of Manitoba Winnipeg Manitoba Canada; ^2^ Institute of Biodiversity Animal Health & Comparative Medicine University of Glasgow Glasgow UK; ^3^ Cape Eleuthera Institute Rock Sound Bahamas; ^4^ VEMCO Ltd. Bedford Nova Scotia Canada

**Keywords:** acoustic telemetry, animal tracking, fine‐scale positioning, fish behaviour, fish movement, high residence 2 receiver (HR2), VEMCO positioning system

## Abstract

1. Acoustic telemetry is an important tool for studying the behaviour of aquatic organisms in the wild.
2. VEMCO high residence (HR) tags and receivers are a recent introduction in the field of acoustic telemetry and can be paired with existing algorithms (e.g. VEMCO positioning system [VPS]) to obtain high‐resolution two‐dimensional positioning data.
3. Here, we present results of the first documented field test of a VPS composed of HR receivers (hereafter, HR‐VPS). We performed a series of stationary and moving trials with HR tags (mean HR transmission period = 1.5 s) to evaluate the precision, accuracy and temporal capabilities of this positioning technology. In addition, we present a sample of data obtained for five European perch *Perca fluviatilis* implanted with HR tags (mean HR transmission period = 4 s) to illustrate how this technology can estimate the fine‐scale behaviour of aquatic animals.
4. Accuracy and precision estimates (median [5th–95th percentile]) of HR‐VPS positions for all stationary trials were 5.6 m (4.2–10.8 m) and 0.1 m (0.02–0.07 m), respectively, and depended on the location of tags within the receiver array. In moving tests, tracks generated by HR‐VPS closely mimicked those produced by a handheld GPS held over the tag, but these differed in location by an average of ≈9 m.
5. We found that estimates of animal speed and distance travelled for perch declined when positional data for acoustically tagged perch were thinned to mimic longer transmission periods. These data also revealed a trade‐off between capturing real nonlinear animal movements and the inclusion of positioning error.
6. Our results suggested that HR‐VPS can provide more representative estimates of movement metrics and offer an advancement for studying fine‐scale movements of aquatic organisms, but high‐precision survey techniques may be needed to test these systems.

## 1 INTRODUCTION

Recent advances in biotelemetry have revolutionized the scales at which aquatic organisms can be monitored in the wild, with data on the locations of individuals being collected more frequently and over larger geographic areas than previously possible (Baktoft et al., 2015; Binder, Holbrook, Hayden, & Krueger, 2016; Biesinger et al., 2013; Cooke et al., 2013). One area that has seen dramatic advancement has been the use of acoustic telemetry to gain accurate estimates (within a few meters) of the two‐dimensional (2D) positions of aquatic organisms tagged with acoustic tags (Binder et al., 2016). Acoustic telemetry positioning systems typically consist of several stationary receivers arranged in a regularly spaced array of near equilateral triangles or squares with overlapping detection ranges. The positions of tagged individuals can then be calculated using the detection data collected from all receivers within the array based on time difference of arrival (TDOA) methodology (Biesinger et al., 2013; Espinoza, Farrugia, Webber, Smith, & Lowe, 2011; Smith, 2013). While the techniques and algorithms for transforming raw detection data to 2D positions are now well‐established, the capability to obtain high‐resolution temporal positional data over a large area (e.g. an entire lake) remains restricted due to inherent limitations of tag and receiver technologies and costs. For example, previous technologies with high temporal resolution capabilities required the use of cables to attach receivers to shore‐based stations to fulfil power and clock synchronization requirements needed for a positioning system.

The VEMCO positioning system (VPS) is a commonly used acoustic telemetry positioning algorithm that is based on a proprietary pulse‐position modulation (PPM) coding scheme (69 and 180 kHz; VEMCO Ltd., Bedford, Nova Scotia, Canada) (Biesinger et al., 2013; Espinoza et al., 2011; Smith, 2013). Despite its regularity of use, the PPM coding scheme has some disadvantages that limit its ability to estimate high‐resolution positional data to within seconds. PPM tags require a few seconds to transmit (Meckley, Holbrook, Wagner, & Binder, 2014), with a receiver having to detect all the transmission pulses, without interference from other tags, to properly decode the tag ID. For instance, if a single tag with a burst length (i.e. time to transmit the pulses) of 3 s and transmission delay (i.e. time between transmissions) of 5 s was in a VPS, the shortest possible positioning period is 8 s. However, when two or more tags are present, it is possible for transmissions to collide and not be detected, so transmission delay must be chosen to keep the collision rates at an acceptable level. Because of this, typical PPM VPS transmission periods exceed 1 min or longer. Moreover, this adverse effect of transmission interference on tag identification generally results in a positive relationship between tag transmission period and the number of tags being successfully detected by a receiver, thus limiting the temporal resolution of positioning using PPM VPS and/or the number of organisms that can be tracked in a small area.

The recent introduction of VEMCO high residence (HR) tags and receivers allows for the monitoring of aquatic animals at a temporal resolution <1 s, while maintaining traditional 180 kHz PPM technology. The HR tags emit a very short (<10 ms) transmission with its ID encoded that HR receivers can decipher, allowing for more tags to be detected with higher temporal resolution than PPM tags. As a result, when used with VPS algorithms, HR technology should allow researchers to monitor the spatial movements of many aquatic animals within a small area at high temporal resolution, with reduced collisions, thereby greatly expanding the ability to study the behaviour of many organisms simultaneously.

Here, we present results of the first documented field test of a VPS composed of HR receivers (hereafter, HR‐VPS). We performed a series of stationary and moving trials with HR tags to evaluate the precision, accuracy and temporal capabilities of this technology. In addition, we present a sample of data obtained for five European perch *Perca fluviatilis* implanted with HR tags to illustrate how this technology can estimate the fine‐scale behaviour of aquatic organisms.

## 2 MATERIALS AND METHODS

### 2.1 Study site

**Figure 1:**
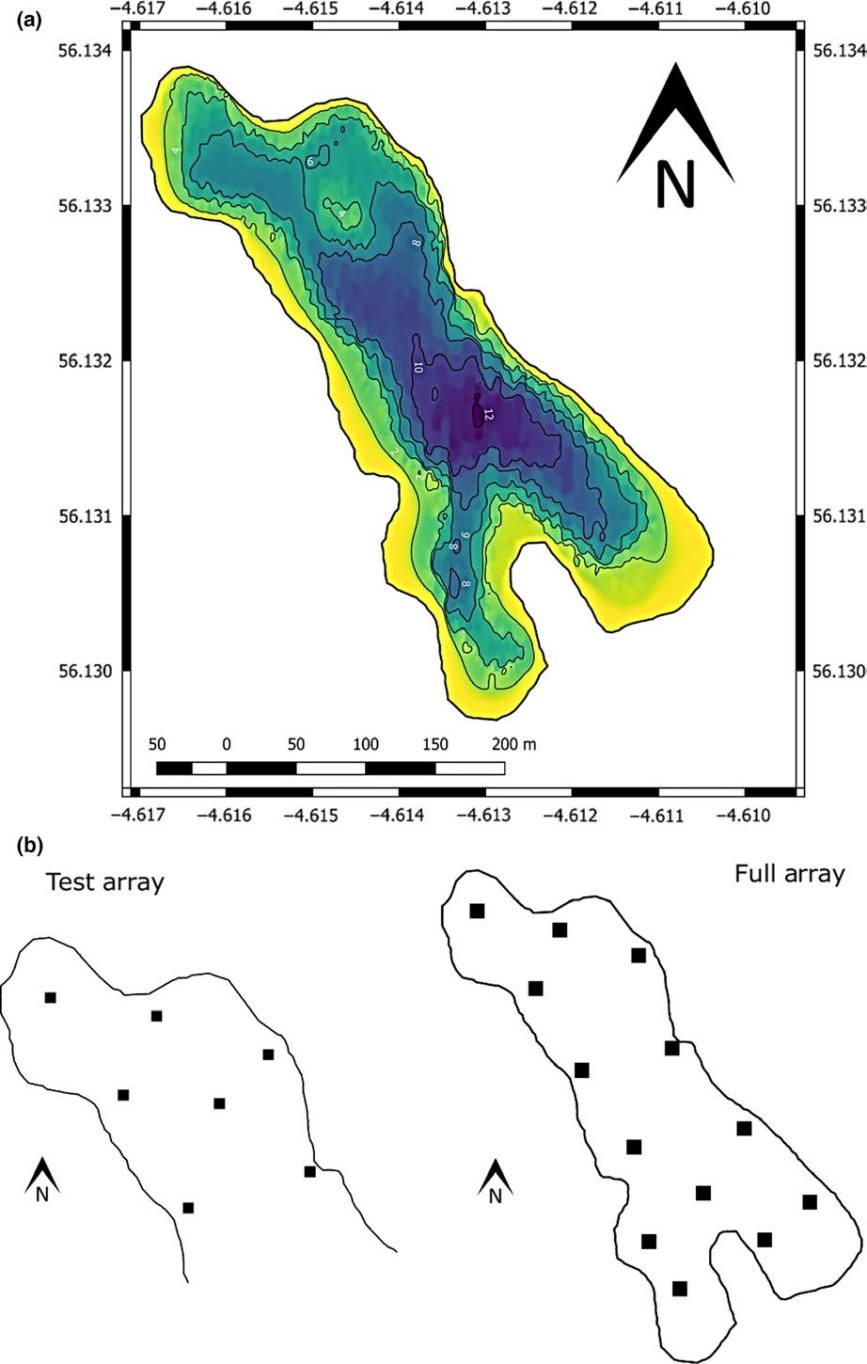
(a) Bathymetry map of the Dubh Lochan, Scotland, UK (56.13N, −4.61W) and (b) maps indicating the locations of high residence 2 receivers making up the test array (*n* = 7 receivers) used in the stationary and movement trials and the full array (*n* = 13 receivers) used to track the movement of European perch as part of the larger study for which this equipment was tested

The Dubh Lochan is a natural, small (surface area = 10 ha), shallow (mean depth = 5 m), lowland freshwater lake located in Scotland, UK (Figure 1a). The lake has a fine sediment substratum and its perimeter is surrounded by a 1–2 m boundary of macrophytes which rise from the bottom of the lake extending above the surface. During the study, the Secchi depth was on average 2 m and water temperatures, measured by sensors co‐located on the acoustic receivers, which were set at various depths within the lake, averaged 10°C. The lake is closed to the public, with fishing and boating not permitted greatly reducing potential issues of surrounding noise on detection efficiency.

### 2.2 Stationary and movement trials

Our test array consisted of seven underwater omnidirectional HR acoustic receivers (high residence 2 receiver 180 kHz; VEMCO Ltd.) positioned 77.1 ± 18.6 m (*M* ± *SD*) apart from each other with overlapping detection ranges (determined by a range test using our test tags, see Supporting Information) covering the northwest half of the lake (Figure 1b). Receivers were mounted pointing upwards to vertical aluminium rods, which were fixed in 20 kg buckets of concrete with two crossed steel rods at the base and deployed at the bottom the lake. Receivers and the GPS (Garmin GPS Map 60CSx; Garmin Ltd., Kansas City, Ohio, USA) used in the study were synchronized to comparable timestamps. Internal clocks of the receivers were synchronized using internally co‐located transmitters (“sync tags”). The test tags used for range testing and stationary and movement trials were V5‐HRs (180 kHz, VEMCO Ltd.) with a mean HR transmission period of 1.5 s (range 1–2 s).

To compare stationary position estimates of the HR‐VPS with GPS‐recorded locations of the test tags, we deployed three tags at haphazard locations throughout the positioning system at depths of 1–2 m (Table 1, Figure 1b). Tags were deployed separately at known locations each on a separate weighted anchor line, with a buoy extending to the surface. This procedure was repeated four times yielding 12 stationary positions for comparison. Position and deployment/recovery times were recorded for each stationary tag using the GPS (Table 1).

**Table 1:** Summary of stationary (S) trials, including the duration (nearest minute), number of positions estimated by HR‐VPS and the median [5th–95th percentiles] and range of accuracy and precision estimates

Trial ID	Duration (min)	No. of estimated positions	Accuracy (m)^a^		Precision (m)^b^	
			Median [5th–95th percentile]	Range	Median [5th–95th percentile]	Range
S1	37	1,443	5.6 [5.3–5.7]	4.5–6.7	0.2 [0.01–1.3]	0.002–1.7
S2	48	1,893	4.3 [4.2–4.3]	3.5–4.4	0.1 [0.02–0.1]	0.002–1.2
S3	41	1,567	5.6 [5.5–5.7]	4.9–5.8	0.1 [0.01–0.2]	0.002–1.2
S4	30	1,166	4.6 [4.5–4.7]	4.3–4.9	0.04 [0.01–0.24]	0.002–0.6
S5	30	1,165	7.7 [7.6–7.8]	7.4–8.7	0.1 [0.04–0.3]	0.005–2.4
S6	37	1,404	5.7 [5.5–5.8]	5.0–7.6	0.1 [0.03–0.5]	0.004–3.5
S7	39	782	7.5 [7.0–8.0]	6.8–8.5	0.4 [0.05–1.0]	0.01–1.3
S8	31	1,167	9.7 [9.4–10.3]	7.9–20.3	0.2 [0.1–6.3]	0.01–16.6
S9	45	1,578	10.8 [10.2–11.6]	9.5–12.0	0.2 [0.04–1.3]	0.001–1.9
S10	52	5	7.3 [7.3–7.4]	7.3–7.4	0.1 [0.04–0.1]	0.04–0.1
S11	41	1,323	5.1 [4.8–5.4]	4.6–7.9	0.2 [0.04–0.6]	0.001–4.1
S12	34	458	2.7 [2.2–2.9]	2.0–7.0	0.2 [0.1–1.2]	0.003–5.6

GPS accuracy was ±3 m (50% CEP) during the trials.

HR‐VPS, high residence‐VEMCO positioning system; CEP, circular error probable.

a Estimated as the distance between each position estimated by HR‐VPS from GPS recorded position for that trial.

b Estimated as the distance between each position estimated by HR‐VPS from the median position estimated by HR‐VPS for that trial.

**Figure 2:**
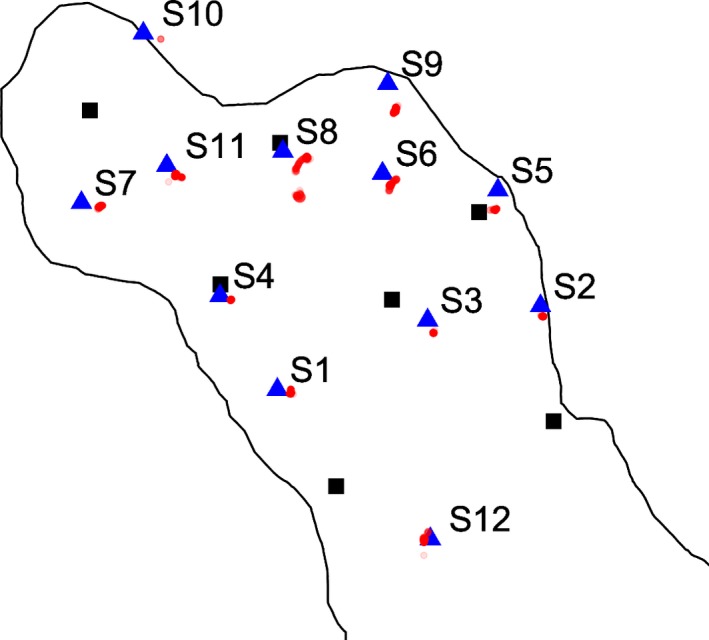
Results of stationary tag trials (S1–S12), including the GPS recorded position of the tag for each trial (blue triangle), all positions estimated by the high residence‐VEMCO positioning system algorithm (red dots), and the location of the receivers in the array for reference (black squares). Estimated positions are semi‐transparent to allow denser regions of position accumulation to opaquer. Note, S10 was placed inside of the thick macrophyte lining the edge of the lake, to which the outline of the lake corresponds

To compare moving tracks between the HR‐VPS and the GPS, a V5‐HR tag was placed on a weighted line 1–2 m below the water surface and towed using a boat outfitted with an electric motor. Moving tests were conducted for 10 min, with five tests total and were spread throughout the array (Table 2, Figure 2). Time and positions of the moving test were recorded every second by the GPS that was held above the tag during movement.

**Table 2:** Summary of movement (M) trials including the duration (nearest minute), number of positions estimated by HR‐VPS and the median [5th–95th percentiles] and range of accuracy estimates

Trial ID	Duration (min)	No. of positions	Accuracy (m)^a^	
			Median [5th–95th percentile]	Range
M1	11	357	4.9 [1.5–9.2]	0.2–10.8
M2	13	381	11.9 [3.6–20.1]	2.1–25.2
M3	12	311	6.3 [4.9–9.2]	3.6–17.0
M4	12	362	10.4 [5.0–15.8]	2.4–19.4
M5	12	391	11.8 [4.7–14.8]	3.0–36.1

HR‐VPS, high residence‐VEMCO positioning system.

a Estimated as the distance between each position estimated by HR‐VPS from the time‐matched position on the GPS track.

Following receiver recovery and downloads, raw data were processed by VEMCO into 2D positions of each test tag using hyperbolic positioning algorithms based on TDOA for each acoustic transmission detected by three or more receivers in the array (Espinoza et al., 2011; Smith, 2013). When a transmission is detected by three or more receivers, a position is calculated using every subset of three receivers, with a single position calculated on a weighted mean that favours the lowest error sensitivity (Smith, 2013). GPS records of tag position were not provided to VEMCO and were therefore compared to HR‐VPS estimate positions independently. The accuracy of the GPS to record receiver and tag locations during the field tests was displayed as ±3 m, but the exact meaning of this error estimate (e.g. 50% confidence or 95% confidence) is not documented by Garmin.

The positional dataset obtained from HR‐VPS analysis by VEMCO for stationary and moving tests performed had 16,856 positions. The recorded start and end times and tag IDs for each stationary and movement trial were used to split the dataset into 17 subsets to assess each trial individually (Tables 1, 2). No data quality filters were applied to the positions obtained by the HR‐VPS prior to analysis. For each stationary trial, we calculated the accuracy of the HR‐VPS as the distance in metres between each estimated position and the recorded GPS position using Pythagorean Theorem. The precision of the HR‐VPS positioning for the stationary tests were assessed by calculating the median position of all estimated HR‐VPS positions for each trial and calculating the distance of each estimated HR‐VPS position from this median position (Table 1). For moving trials, accuracy was assessed as the distance between each estimated position and the time‐matched recorded GPS position. We then calculated the median, 5th and 95th percentiles and range of the accuracy and precision estimates for both stationary and moving trials (Tables 1, 2).

### 2.3 Fish movement data

Following field testing of the HR‐VPS, the acoustic array was extended to cover the entire area of the lake (Figure 1b). We captured 26 European perch *Perca fluviatilis* between 27 July 2016 and 14 September 2016 with mean (±*SD*) fork length and mass of 232.4 ± 32.1 mm and 198.1 ± 70.6 g respectively. Each captured fish was surgically implanted with an HR acoustic tag (V9‐HR, 180 kHz, VEMCO Ltd.) with a mean HR transmission period of 4 s (range 3–5 s). All fish were released in the middle of the study lake on 20 September 2016.

Position data, estimated using VPS algorithms as described above, from a random 15‐min period on 2 November 2016 were selected from a random subset of five perch to test the effect of transmission period on movement metric estimation. When then thinned the positional data from each of the five fish to have minimum transmission periods of 7, 10, 15, 30, 45 and 60 s, giving a total of seven positional datasets for each fish (the original HR‐VPS dataset and the six thinned datasets). Perch position data were thinned using a function written in the statistical package r that calculated the transmission period for all positions for a given trial and then removed the first (chronologically) position that had a transmission period from the preceding position that was less than a set time. Next, it would again recalculate all transmission periods and delete the first one that was less than that set time. This process was repeated until the new dataset contained only positions that had transmission periods that were less than the set time. For each fish/dataset, we calculated the following common movement metrics: total distance travelled, mean turning angle and mean speed. We then used linear regression to determine how estimates of the movement metrics changed as a function of transmission period.

## 3 RESULTS

### 3.1 Stationary and movement trials

The median precision (95th percentile interval) of all HR‐VPS estimates from all stationary trials (*n* = 12) was 0.1 m (0.02–0.07 m), with individual precision estimates ranging from 0.001 m to 16.7 m depending on the trial. S4 had the highest median precision at 0.04 m (0.01–0.2 m) and deployment S7 had the lowest highest precision 0.4 m (0.04–1.0 m) (Table 1, Figure 2). The median accuracy (95th percentile interval) of HR‐VPS estimates from all stationary trials (*n* = 12) was 5.6 m (4.2–10.8), with individual accuracy estimates for individual position ranging from 2.0 to 20.3 m depending on the trial. Trial S12 was most accurately positioned (2.7 m [2.2–2.9 m]) and S9 was the least accurately positioned (10.8 m [10.2–11.6 m]) (Table 1, Figure 2). Most positions estimated by the HR‐VPS were positioned to the southeast of the GPS‐recorded position for a given stationary test location suggesting a positioning bias between HR‐VPS and the GPS, although this was not the case for S9 and S12, where HR‐VPS positions were to the west and southwest of the GPS location respectively (Figure 2). Of interest was trial S10, which was purposely placed into thick macrophyte at an approximate depth of 1.5 m. S10 was only positioned five times by the HR‐VPS; however, these position estimates were still precise (0.08 m [0.04–0.1 m]) and quite accurate (7.3 m [7.3–7.4 m]) (Table 1, Figure 2).

**Figure 3:**
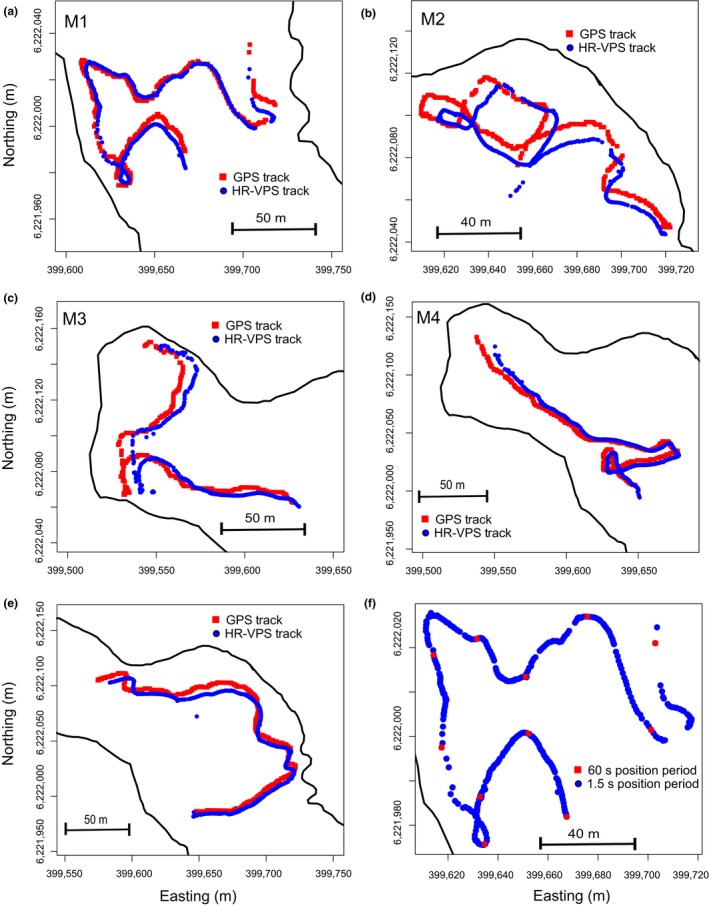
(a–e) Results of moving tag trials (M1–M5) indicating the GPS track recorded at 1 s intervals (blue squares) and the track estimated by the high residence‐VEMCO positioning system (HR‐VPS) algorithm (red dots). Corresponding values of distances between HR‐VPS estimated and GPS positions are provided in Table 3. (f) Example of a movement track (M1) from an HR tag using the HR‐VPS positions obtained from the native (mean 1.5 s) transmission period thinned to simulate a tag with a 60 s transmission period

The median (±95th percentile interval) accuracy of HR‐VPS estimates for all moving trials (*n* = 5) was 8.83 ± 2.85 m with moving test M1 being most similar (4.9 m [1.5–9.2 m]) and M2 the least (11.9 m [3.6–20.1 m]) (Table 2, Figure 3). Tracks produced by HR‐VPS closed matched those of the GPS recording the position of the tag during each movement trial, but like stationary trials seemed to be positioned to the southeast of the GPS track in many cases (Figure 3). When the transmission period of a moving track produced by the HR‐VPS was reduced to 60 s and compared to the original track from the HR tag with transmission period of 1.5 s, clear omissions in movements were observed (Figure 3f).

### 3.2 Fish movement data

**Figure 4:**
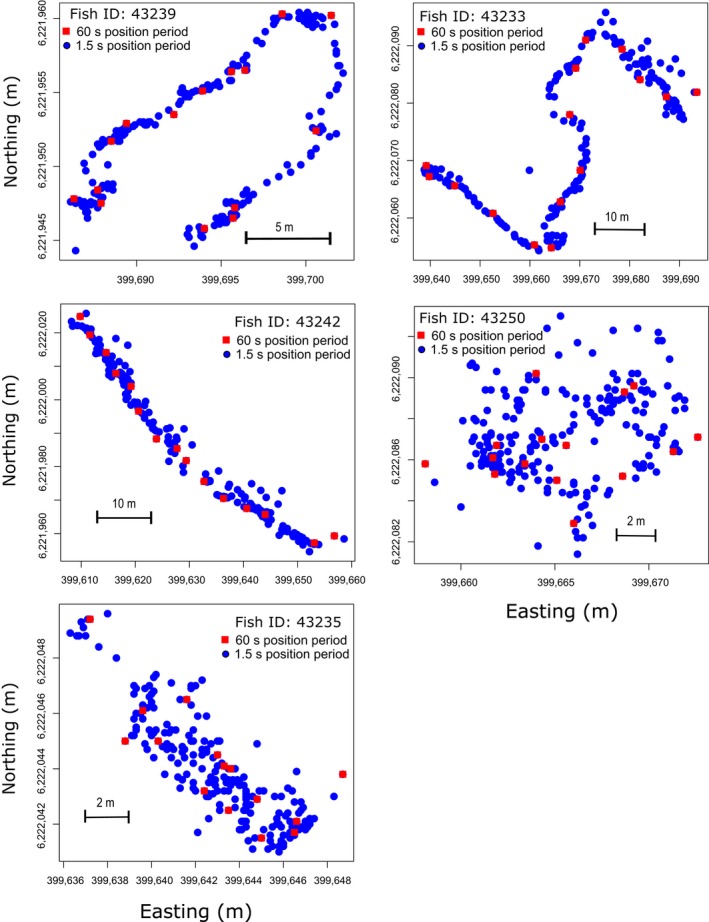
Comparison of movement tracks of five perch implanted with high residence tags with mean transmission period of 4 s (blue circles) relative to the same tracks thinned to simulate a 60 s transmission period (red squares)

**Figure 5:**
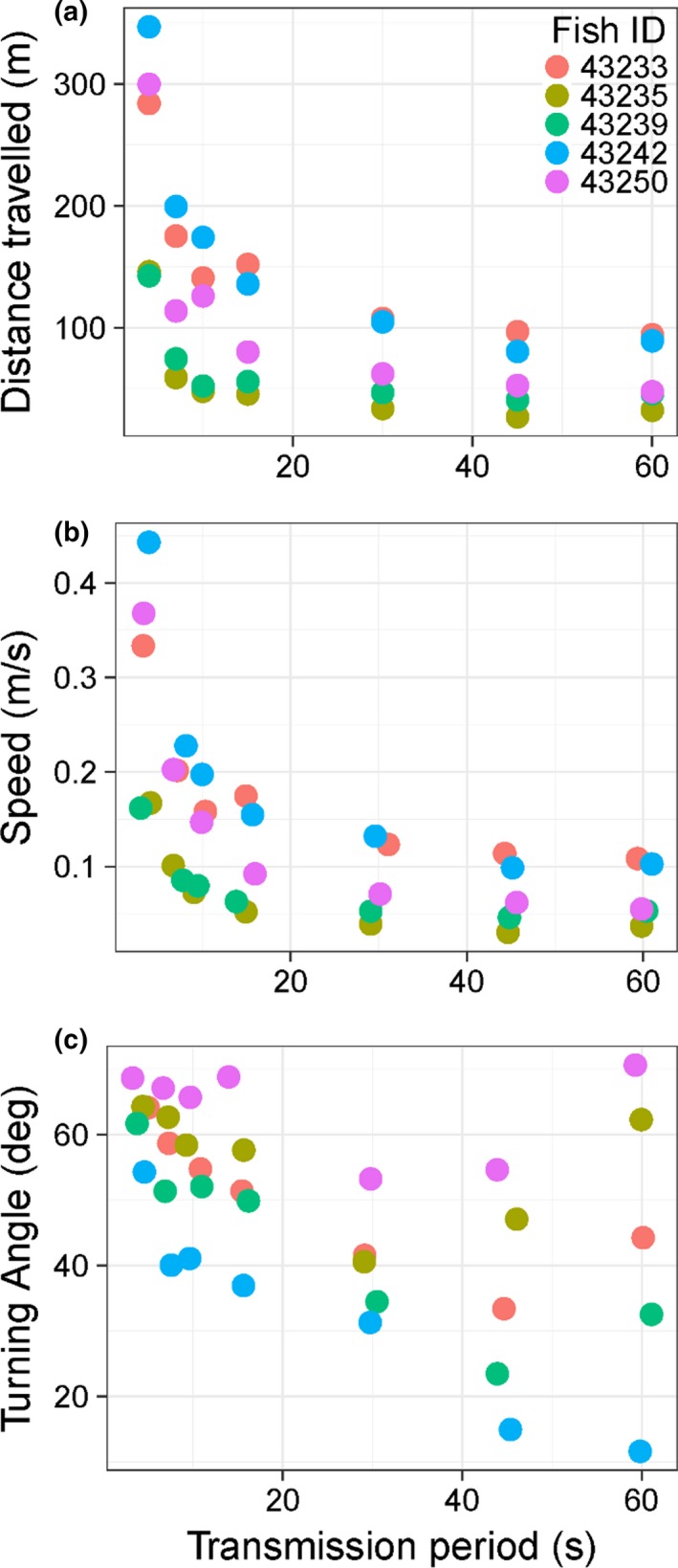
Comparison of estimates of common movement metrics calculated at various transmission periods for the movement tracks of each fish presented in Figure 4, including (a) total distance travelled, (b) mean speed and (c) mean turning angle. The transmission period of 4 s is the mean transmission period of the high residence tag implanted in the fish. Transmission period of 7, 10, 15, 30, 45 and 60 s were obtained by degrading the data from the fish data. Note that points are slightly offset on the *x*‐axis at each transmission period to prevent overlap of data points

A total of 1,051 positions (range 180–223 positions/fish) were obtained for the 15 min subsets of the five‐tagged perch. Comparisons between movement metrics estimated for the subset perch data using the full HR‐VPS array and HR tag (mean HR transmission period = 4 s) compared to this data thinned to represent older VPS PPM technology with a transmission period of 60 s resulted in a 93% reduction in the number of positions estimated. As a result, estimates of movement metrics also varied with transmission period. Distance travelled, speed of movement and turning angle for fish implanted with HR tags each decreased with longer transmission period (log_10_ distance travelled: *F*_1,33_ = 16.2, *p* < .01, *r* ^2^ = .33, *y* = −0.01*x* + 2.14); (log_10_ speed of movement: *F*_1,33_ = 20.55, *p* < .01, *r* ^2^ = .38, *y* = −0.01*x* − 0.76); (turning angle *F*_1,33_ = 10.59, *p* < .01, *r* ^2^ = .24, *y* = −0.37*x* + 58.49). However, the relationship was much more variable for turning angle than estimates for distance travelled and speed of movement, particularly at the slowest transmission frequencies (Table 3, Figures 4, 5). Mean turning angles also varied among fish, with those fish (IDs 43235 and 43250) with less linear, more erratic movement tracks having their turning angles from simulated transmission frequencies of 60 s overestimated (Table 3, Figures 4, 5).

**Table 3:** The effect of tag transmission period on observed positioning period, the number of VPS positions obtained for five perch implanted with HR tags and on the estimation of common movement metrics, including distance travelled (m) and median (5th–95th percentile) of speed (m/s) and turning angle (°) estimated for each fish

Metric	Transmission period (s)^a^	Position period (s)^b^	No. of positions	Fish ID				
				43239	43233	43242	43250	43235
Distance travelled	HR tag, 4	4.2 (4.0–5.0)	215 (180–223)	142.6	284.0	346.8	299.8	145.9
	7	8.5 (8.2–9.4)	95 (72–106)	74.5	175.2	199.4	113.8	59.1
	10	12.0 (11.7–13.1)	68 (57–75)	52.2	140.9	174.1	126.0	47.9
	15	16.6 (16.3–17.9)	54 (50–55)	55.8	151.9	135.9	80.2	45.6
	30	31.9 (31.4–34.6)	28 (24–29)	46.8	107.6	104.8	62.2	33.8
	45	47.2 (46.6–48.4)	19 (18–20)	40.8	96.8	80.5	52.7	26.8
	60	61.9 (61.6–62.4)	15 (15–15)	45.9	94.3	89.2	47.6	32.3
Speed	HR tag, 4	4.2 (4.0–5.0)	215 (180–223)	0.13 (0.03–0.35)	0.23 (0.05–0.99)	0.25 (0.08–1.55)	0.22 (0.05–0.99)	0.13 (0.02–0.45)
	7	8.5 (8.2–9.4)	95 (72–106)	0.06 (0.01–0.18)	0.16 (0.03–0.50)	0.14 (0.06–0.89)	0.15 (0.04–0.46)	0.08 (0.02–0.22)
	10	12.0 (11.7–13.1)	68 (57–75)	0.06 (0.01–0.15)	0.15 (0.04–0.32)	0.12 (0.05–0.65)	0.10 (0.04–0.32)	0.07 (0.02–0.15)
	15	16.6 (16.3–17.9)	54 (50–55)	0.06 (0.01–0.14)	0.15 (0.05–0.37)	0.11 (0.05–0.44)	0.07 (0.02–0.21)	0.04 (0.01–0.11)
	30	31.9 (31.4–34.6)	28 (24–29)	0.04 (0.02–0.11)	0.14 (0.03–0.19)	0.11 (0.06–0.27)	0.06 (0.02–0.15)	0.04 (0.01–0.06)
	45	47.2 (46.6–48.4)	19 (18–20)	0.04 (0.01–0.10)	0.11 (0.05–0.17)	0.10 (0.06–0.14)	0.06 (0.02–0.10)	0.03 (0.01–0.06)
	60	61.9 (61.6–62.4)	15 (15–15)	0.04 (0.02–0.11)	0.11 (0.05–0.16)	0.09 (0.06–0.18)	0.05 (0.01–0.12)	0.03 (0.02–0.07)
Turning angle	HR tag, 4	4.2 (4.0–5.0)	215 (180–223)	65.3 (3.29–130.0)	66.1 (1.74–141.2)	56.3 (5.22–102.3)	76.2 (6.93–124.5)	63.1 (5.43–127.9)
	7	8.5 (8.2–9.4)	95 (72–106)	35.9 (2.91–128.3)	38.6 (3.39–144.1)	31.2 (2.77–96.5)	67.5 (5.23–132.1)	62.9 (11.7–120.7)
	10	12.0 (11.7–13.1)	68 (57–75)	47.7 (1.97–130.0)	28.8 (1.68–152.9)	28.7 (3.72–97.5)	64.1 (3.57–148.8)	55.8 (11.5–114.9)
	15	16.6 (16.3–17.9)	54 (50–55)	39.7 (2.89–137.0)	28.7 (4.66–160.1)	26.8 (3.00–94.8)	63.6 (4.3–150.5)	49.7 (8.65–137.1)
	30	31.9 (31.4–34.6)	28 (24–29)	19.1 (4.23–106.4)	18.3 (3.16–143.8)	21.6 (3.00–101.1)	32.4 (4.82–150.0)	34.1 (3.45–123.8)
	45	47.2 (46.6–48.4)	19 (18–20)	11.8 (1.63–63.1)	9.54 (1.39–140.2)	11.2 (4.02–47.5)	36.4 (1.71–136.0)	23.3 (9.14–143.4)
	60	61.9 (61.6–62.4)	15 (15–15)	27.1 (6.32–71.3)	13.7 (4.38–148.9)	6.36 (1.43–34.1)	54.6 (7.43–139.6)	33.6 (5.23–159.8)

HR, high residence; VPS, VEMCO positioning system.

a Transmission period represents the theoretical interval a tag would transmit a coded signal for positioning; however, because not all transmissions are detected or positioned, this value represents the minimum transmission period achievable. The HR tags had a mean transmission period of 4 s. Transmission frequencies of 7–60 s were obtained by thinning the fish HR data (see Materials and methods).

b Values are the median (range) of the five fish.

## 4 DISCUSSION

The HR‐VPS was able to position a stationary and moving tag to within a few meters in most cases with high temporal resolution making it ideal for determining the fine‐scale positioning of aquatic organisms. The ability to accurately estimate position on a per second basis with negligible signal collisions allows positioning of high numbers of tags at the same time making it ideal for studying organisms in areas where there are migration bottlenecks or aggregations for events including reproduction and foraging. HR‐VPS will also facilitate the analysis of behaviour in wild aquatic organisms at temporal resolutions not previously possible using independent receivers. However, limitations such as small spatial scales due to small detection range of 180 kHz receivers and the cost of multiple receivers needed in a HR‐VPS array should be considered.

We found HR‐VPS to have median positioning accuracy in stationary trials ranging 2.7–10.8 m, as measured by the handheld GPS. However, we suspect that HR‐VPS was indeed more accurate than the values reported here for several reasons. First, the strong southeastern bias advises that the calibrated positions of the receivers are very accurate relative to each other, as indicated by the high precision of positions for stationary trials. This suggests that if a high‐accuracy GPS were used to survey the system, the results would be both precise (difference of HR‐VPS positions relative to one another) and accurate (i.e. difference between positions estimated by HR‐VPS and recorded by GPS). It is unclear what contributed to the consistent southeastern error skew in our study between handheld GPS and HR‐VPS estimates. One possibility could be error in the handheld GPS measurements of the receiver locations, as only a single GPS position was recorded for each tag location during stationary trials and the displayed GPS accuracy was 3 m. In fact, the exact meaning of the displayed 3 m error is not specified by GPS manufacturer and may be as low as 50% CEP (CEP [circular error probable]), meaning that 50% of all measurements would be within a radius of 3 m and 95% CEP would be within 6 m (Misra & Enge, 2010). Moreover, because the HR‐VPS analysis uses sync tag data to measure distances between neighbouring receivers, it is likely that the HR‐VPS positions are more accurate in a relative sense with respect to one another, but not necessarily with respect to their actual locations on the earth measured by the handheld GPS through the triangulation of satellites. A source of error in the movement trials could be the movement of the tag under and to the side of the boat during the movement tests; thus, causing a small discrepancy in the spatial locations of the GPS and the transmitting tag, although the magnitude of error stemming from this factor is difficult to determine. Whatever the case, these results suggest that estimated position accuracy will only be as good as the accuracy of the GPS coordinates for receivers and as advancements in telemetry continue to progress more accurate surveying techniques for determining receiver deployment locations will become necessary.

Although our results demonstrate that the HR‐VPS system can provide positional data at unprecedented temporal scales, there are some areas that require further study to determine the limitations of the system and the degree of advance beyond previous technologies. For example, it would be useful to test the system over a range of environmental conditions including environments with differing bathymetry, macrophyte abundance, current speeds, ice conditions and many other abiotic factors, as similar factors have also been found to impact detection efficiency for PPM acoustic technology (e.g. Huveneers et al., 2016; Steel, Coates, Hearn, & Klimley, 2014). For example, stationary test S10 was purposefully placed within a surrounding mass of macrophytes and resulted in very few useable detections by our positioning system, indicating that the HR‐VPS positioning system may not be suitable for monitoring aquatic organisms that frequently use or spend long periods of time under dense cover. Furthermore, and irrespectively of absolute position accuracy, our results show that such a high‐resolution tracking system is capable of accurately reflecting complex path tortuosity (Figure 3f). Tortuosity is an important feature of movement (Benhamou, 2004) that remains difficult to encapsulate using classical low‐resolution tracking technologies, despite the immense improvements made in animal movement modelling in the last years (Hooten, King, & Langrock, 2017). Encapsulating the natural tortuosity of an animal's movements using high‐resolution tracking technology can in fact contribute, to provide appropriate estimates of trajectory characteristics (i.e. bearings and speed for continuous‐time models or turning angles and step length for discrete‐time models) to be used, in combination with environmental covariates, to depict existing behavioural modes (also called behavioural states or processes) and switches between behavioural modes (Parton & Blackwell, 2017). The downside of increasing spatio‐temporal resolution of animal tracks becomes, however, apparent when step length (distance between successive positions) becomes smaller or equivalent to positioning error, generating jagged movement tracks (see Figure 4) and thus inflating measured movement metrics, such as distances travelled. Based on our results, we can conclude that the tested technology represents a unique opportunity to provide improved movement data and behavioural information on free‐ranging animals, under the condition that appropriate modelling methods are employed to capture behavioural modes and switches occurring at different spatio‐temporal scales and overcoming problems related to sampling rates (Baktoft, Gjelland, Økland, & Thygesen, 2017; Baktoft et al. 2015; Calabrese, Fleming, & Gurarie, 2016; Fleming et al., 2014; Pedersen, Righton, Thygesen, Andersen, & Madsen, 2008).

The study of individual variation in behavioural and physiological traits has experienced a surge of interest over the last decade, with much of this work being done on aquatic organisms and especially fish (Burton, Killen, Armstrong, & Metcalfe, 2011; Killen, Marras, Metcalfe, McKenzie, & Domenici, 2013; Metcalfe, Van Leeuwen, & Killen, 2016). However, we still have very little understanding of the ecological consequences of inter‐individual phenotypic variation due to our limited ability to measure the movements of organisms in the natural aquatic environment. Our results suggest that the HR‐VPS technology should close this critical gap, finally providing accurate measures of spontaneous activity, foraging ability and habitat preferences in the wild. This will allow individual variation in traits such as metabolic rate, stress responsiveness and personality of multiple individuals in a confined area to be directly related to movement patterns (migration, foraging habits and spawning aggregations) in free‐ranging animals (Baktoft et al., 2016; Laskowski et al., 2016; Treberg, Killen, MacCormack, Lamarre, & Enders, 2016). From a conservation perspective, this technology will facilitate the study of how animal movements change in response to natural and anthropogenic change in variables such as temperature, oxygen and food availability. Furthermore, the direct behavioural responses of aquatic animals to human activities such as boat noise (Simpson et al., 2016), ecotourism (Heyman, Carr, & Lobel, 2010) and fishing pressure (Tsuboi, Morita, Klefoth, Endou, & Arlinghaus, 2015) can be more accurately assessed.

## AUTHORS' CONTRIBUTIONS

M.M.G., T.E.V.L., J.H., M.N. and B.K. performed field work; D.M.W. and F.I.S. helped with the study design and interpretation of results; M.M.G., T.E.V.L. and S.S.K. performed data analysis; M.M.G., T.E.V.L. and S.S.K. wrote the initial draft. All authors contributed to the writing and editing of the paper.

## DATA ACCESSIBILITY

The data reported in this paper have been deposited in the open source database Zenodo at https://doi.org/10.5281/zenodo.1174331.
