# Supplementary material for: Field testing a novel high residence positioning system for monitoring the fine‐scale movements of aquatic organisms
Source: Methods Ecol Evol. 2018 Mar 24;9(6):1478–88. doi: 10.1111/2041-210X.12993 (PMC6033000; doi:10.1111/2041-210X.12993)
Supplement: Supplementary file 1 [file MEE3-9-1478-s001.docx]

Supplemental Information:

**Field testing a novel high residence positioning system for monitoring the fine-scale movements of aquatic organisms**

Matthew M. Guzzo^1, $, *^, Travis E. Van Leeuwen^2, 3, $^, Jack Hollins^2^, Barbara Koeck^2^, Matthew Netwon^2^, Dale M. Webber^4^, Frank I. Smith^4^, David M. Bailey^2^, Shaun S. Killen^2^

^1^ Department of Biological Sciences, University of Manitoba, Winnipeg, Manitoba, Canada

^2^ Institute of Biodiversity, Animal Health & Comparative Medicine, University of Glasgow, Glasgow, United Kingdom

^3^ Cape Eleuthera Institute, Rock Sound, Eleuthera, Bahamas

^4^ VEMCO Ltd., Bedford, Nova Scotia, Canada

^$^ Authors contributed equally.

***** Corresponding author. mattguzzo12@gmail.com (M.M. Guzzo)

**Range tests**

Range tests were performed prior to setting up the test array and executing stationary and movement tag trials to ensure that the receivers in the array had overlapping detection ranges. Three range tests were performed using a V5-HR tag (180 kHz, VEMCO Ltd., Bedford, NS, Canada) with a mean HR transmission period of 1.5 s (range 1 - 2 s) and an omni-directional high residence acoustic receiver (HR2 180 kHz, VEMCO Ltd.). In each range test the tag was anchored 1 m below the surface of the water for a duration of ≈ 10 minutes. Details on each range test are as follows.

Range Test A: This test took place in the middle of the lake with the receiver anchored a set location and the tag first placed 50 m from the receiver and then moved at 50 m intervals in a straight line from the receiver up to 300 m (Fig. S1).

Range Test B: This test involved keeping the receiver at the same location as in Range Test A but anchoring the tag (i) right in front of the edge of the thick macrophytes lining the edge of the lake, (ii) in the macrophytes, and (iii) in a patch of lily pads located at the edge of the macrophytes (Fig. S1). The tag locations (i-iii) were about 50 m from the receiver for these tests.

Range Test C: This test involved moving the receiver close to the macrophytes near the lakes edge (≈ 5 m) and moving the tag from distances ranging from 5, 25, 50, 75, and 100 m from the receiver at the same distance from the edge of the macrophytes (≈ 5 m) to determine how this factor may impact detection by HR2 receivers (Fig. S1).

The results of these range tests suggest that the unobstructed maximum range of the HR2 with the V5-HR tag in our study lake for near perfect detection efficiency and high signal strength was between 50 and 100 m (Fig. S1). Similar results to the unobstructed range test were found when a range test was performed adjacent to the macrophyte edge. However, when the tag was moved on edge or in the macrophyte or in lily pads, the detection efficiency declined to almost half of that found for a similar distance where the tag was in the open water (Fig. S1).

**
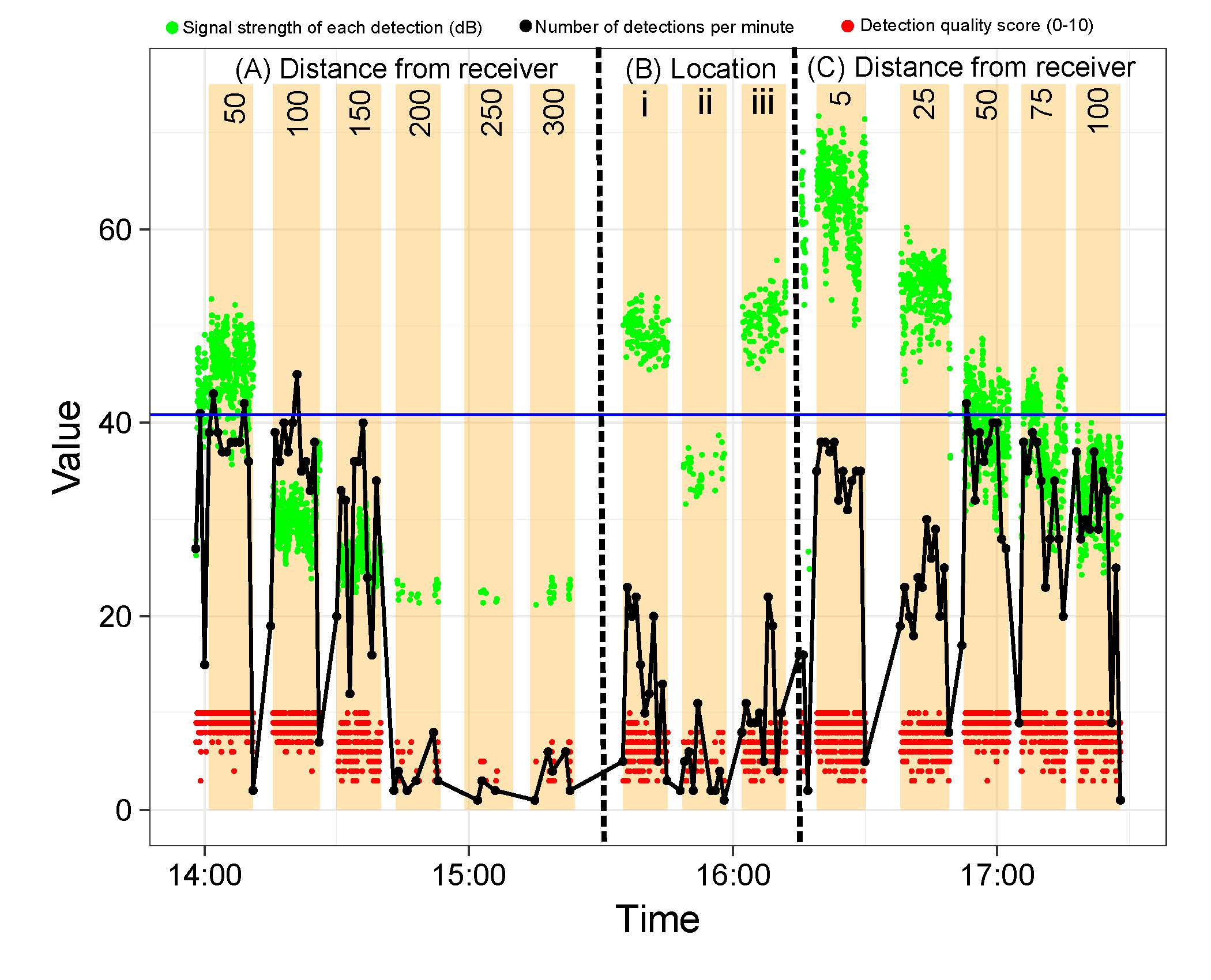
**

**Figure S1.** Results of range testing of a VEMCO HR2 180 kHz receiver and V5-HR 180 kHz tag in the Dubh Lochan, UK. The first range test (A) took place in the middle of the lake with the receiver anchored a set location and the tag first placed 50 m from the receiver and then moved at 50 m intervals in a straight line from the receiver. The second range test (B) involved keeping the receiver at the same location and then moving the tag. The final range test (C) involved moving the receiver close to the macrophytes near the lakes edge and moving the tag from distances ranging from 5, 25, 50, 75, and 100 m from the receiver but near edge of the macrophytes to determine how this factor may impact detection by HR2 receivers. The blue horizontal line indicates 40.85 detections min^-1^, which corresponds to the average number of HR transmissions per minute, so would indicate 100 % detection efficiency.
